# Late periorbital haemorrhage following functional endoscopic sinus surgery: a caution for potential day case surgery

**DOI:** 10.1186/1472-6815-6-11

**Published:** 2006-05-30

**Authors:** Arvind Kumar Arya, David Machin, Hadi Al-Jassim

**Affiliations:** 1 Department of Otolaryngology, Head & Neck Surgery, University Hospital Aintree, Liverpool

## Abstract

**Background:**

Orbital complications following functional endoscopic sinus surgery (FESS) are fortunately rare. They are usually easily and rapidly recognizable.

**Case presentation:**

We present an unusual case of a forty-five year old woman who underwent routine FESS and was not packed nasally after the procedure. Six hours later she started bleeding and nasal packs were inserted. She soon developed unilateral periorbital bruising and within hours her condition had worsened so much that the viability of the eye was thrown into question. She underwent medial and lateral canthotomies and made an uneventful post-operative recovery.

**Conclusion:**

This rare case demonstrates that late, brisk post-operative bleeding can occur after FESS with potentially catastrophic consequences. Clinicians should be aware that discharging patients after FESS too early may lead to medico-legal problems.

## Background

Orbital complications of FESS are fortunately rare [1]. When they do occur, however, the implications can be enormous [2]. Complications reported include orbital plate breach and periorbital haemorrhage, medial rectus muscle damage and optic nerve damage [3].

Late bleeding causing orbital complications following FESS has been reported before [7]. Post-operative bleeding can be classified into arterial (fast) or venous (slow), with the former usually occurring peri- or in the immediate post-operative period. Arterial bleeding is usually from the anterior ethmoidal artery, or rarely from the posterior ethmoidal artery. If the artery is damaged near its entry point in the orbital plate, rapid onset of periorbital ecchymoses and dangerous rising of intra-ocular pressure will occur. In these cases, epistaxis is usually heavy, and ligation of ethmoidal arteries may be required. Venous bleeding causing orbital complications is less likely to cause epistaxis, but rather a slow, progressive periorbital ecchymoses which may take several hours, even days to develop into a dangerous situation. What is unusual in the case to be presented is that there was a delay of four hours before the haemorrhage occurred, and this was likely to be arterial in nature given its rapid progression. Post-operative bleeding following nasal surgery has been reported as being as high as 5.4% but this was in association with a septoplasty being performed [4]. In our case extra-ocular arterial bleeding or bleeding into the maxillary sinus with a breach in the orbital plate may have been dealt with conservatively in principle, but there was little doubt that immediate orbital decompression was warranted. Insertion of a nasal pack to arrest haemorrhage was not desirable, yet necessary. In theory spasm of the artery or lysis of the platelet plug should normally occur, but this evidently was not the case here. The fact that the patient was mobilized early to prepare for going home may have led to the dislodgement of any blood clot, or caused an artery to dilate.

Management of periorbital haematoma depends on the type of haemorrhage (arterial or venous) [8]. Slow venous bleeding should be treated medically with mannitol and steroids, with orbital massage and topical orbital intraorbital pressure (IOP) lowering agents if evidence of raised IOP. Arterial bleeding may be accompanied by epistaxis and this should be controlled with endoscopic arterial ligation/cautery or external (Lynch) frontal ethmoid approach/decompression. Surgical treatment of orbital haematoma includes canthotomies, cantholysis, endoscopic orbital decompression or external frontoethmoid approach. In our case medial and lateral canthotomies were deemed essential to relieve the pressure on the orbit from either side.

Day case surgery has the advantage over in-patient surgery by being cost-effective and resource conserving. It has been promoted by the Department of Health who want 75% of all surgery to be performed in a day case setting by 2010 [5]. This must be weighed up against potential complications, despite being relatively small. It has been suggested that the complication rate of day surgery should be less than 3% [6]. However, if serious late complications such as the one described can occur, there will be medico-legal implications if patients are discharged too early. In our case, the surgery was performed in a centre with no on-call medical staff and no facilities on site for converting day case surgical patients into in-patients. This raises the question whether day case FESS surgery should be performed there, but as this is the first case described of late orbital bleeding, this recommendation should be taken in context with the rarity of the event.

## Case presentation

**Figure 1:**
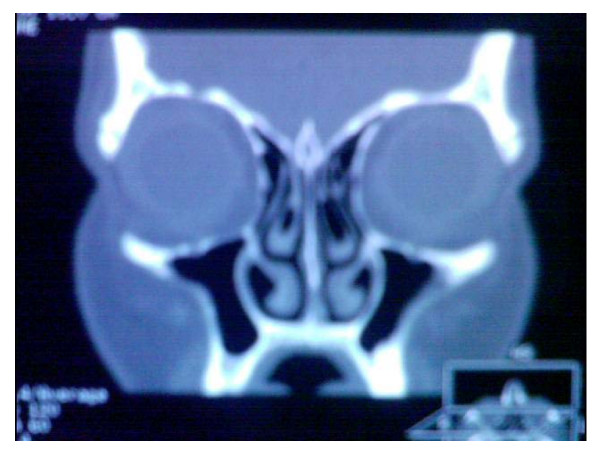
Coronal pre-operative CT scan of the sinuses, demonstrating bilateral concha bellosa and ostiomeatal complex blockage.

Written consent was obtained from the patient for publication of study. This case report reflects on a 45 year old woman who underwent routine functional endoscopic sinus surgery (FESS) for recurrent sinusitis. Pre-operatively she was noted to have bilateral concha bellosa on Computerised Tomography (CT) scanning with subsequent obstruction of both osteo-meatal complexes (see figure 1). Her symptoms were not controlled with medication in the form of steroid sprays and antibiotics and she was therefore listed for FESS.

The operation was performed in a dedicated day case centre with no facilities for in-patient care at a District General Hospital, by an experienced middle grade surgeon. Nasal preparation used was Otrivine 30 minutes beforehand, followed by injection of 2% lignoicaine in a solution of 1:80000 adrenaline into both uncinate processes and middle turbinates. Bilateral uncinectomies with middle meatal antrostomies were performed prior to reduction of both middle turbinates. There was little bleeding noted during the procedure and also after completion of the surgery. Insertion of nasal packs was therefore not deemed to be necessary. She was transferred to the day ward and made a routine immediate post-operative recovery.

Some six hours later she developed brisk bilateral epistaxis, which was uncontrollable with ice and compression for thirty minutes. She required packing with Merocel nasal packs and the active bleeding was arrested. Within an hour of this on further review she developed very slight bruising of the subcutaneous tissues inferior to the left eye. She was not in any discomfort, nor reported any visual disturbance. For completeness the opinion of the duty ophthalmologist was obtained, who reported normal ocular movements and vision. She was transferred to the University Hospital for overnight observations. Prior to transfer the epistaxis was noted to have been stabilized and the periorbital bruising not progressing so she was deemed safe for transfer.

An hour later on arrival at the University Hospital, the bruising had markedly increased and in addition to left infraorbital bruising she had developed swelling of the left eyelid and left circumferential orbital bruising. The nasal packs were removed but the periorbital swelling continued. At this time, some seven hours post-operatively she still had normal eye movements and visual acuity although there was evidence of colour desaturation; not with red but blue and green appeared black. The overriding concern was that for the preservation of the eye and the decision was taken to decompress the orbit. She went immediately to theatre for medial and lateral canthotomies. At surgery haematomas were evacuated particularly laterally and the tenseness of the orbit was markedly reduced. A specific bleeding point was not identifiable. She made an uneventful post-operative recovery. Post-operative CT scan revealed normal ocular anatomy, with no evidence of re-collection of haematoma. Ophthalmological opinion stated no abnormality and orthoptics were normal.

## Conclusion

Surgeons should pay heed to possible late and devastating complications of endoscopic sinus surgery. This should be borne in mind when planning to undertake such surgery as a daycase procedure. Mechanisms must be in place to ensure early identification and treatment of all cases of periorbital haematoma following FESS.

## Competing interests

The author(s) declare that they have no competing interests.

## Authors' contributions

AKA wrote the manuscript, and performed the literature search. HAJ recorded the case history, and contributed to the final manuscript. DM contributed to the literature search and helped write the manuscript.

## Pre-publication history

The pre-publication history for this paper can be accessed here:



## References

[B1] Harkness P, Brown P, Fowler S (1997). A national audit of sinus surgery. Results of the Royal College of Surgeons of England comparative audit of ENT surgery. Clin Otolaryngol.

[B2] Britten N £1.3 m for man brain damaged in surgery on runny nose. http://www.telegraph.co.uk.

[B3] Cumberworth VL, Sudderick RM, Mackay IS (1994). Major complications of functional endoscopic sinus surgery. Clin Otolaryngol.

[B4] (2000). Unexpected overnight admissions following day-case surgery: an analysis of a dedicated ENT day care unit. Ann R Coll Surg Engl.

[B5] (2000). The NHS Plan – A plan for investment, A plan for reform. HMSO, The Copyright Unit, St Clements House, 2-16 Colegate, Norwich NR3 1BQ.

[B6] Hogg RP, Prior MJ, Johnson AP (1999). Admission rates, early readmission rates and patient acceptability of 142 cases of day case septoplasty. Clin Otolaryngol.

[B7] Stankiewicz JA, Chow JM (1999). Two faces of orbital haematoma in intranasal (endoscopic) sinus surgery. Otolaryngol Head Neck Surg.

[B8] Bhatti MT, Stankiewicz JA (2003). Ophthalmic Complicationf of Endoscopic Sinus Surgery. Surv Ophthalmol.

